# Protocol to quantify cell-surface vimentin on senescent human chondrocytes and other cell types via a cell-based ELISA

**DOI:** 10.1016/j.xpro.2026.104451

**Published:** 2026-03-20

**Authors:** Jana Riegger, Hartmut Geiger

**Affiliations:** 1 Division for Biochemistry of Joint and Connective Tissue Diseases, Department of Orthopedics, University of Ulm, 89081 Ulm, Germany; 2 Institute of Molecular Medicine, University of Ulm, 89081 Ulm, Germany

**Keywords:** Cell culture, Cell isolation, Cell Membrane, Flow Cytometry, Immunology, Protein Biochemistry

## Abstract

Surface markers for the identification and potential targeting of senescent cells are limited and typically assessed via flow cytometry. Here, we present a protocol to quantify cell-surface vimentin (CSV) as a membrane-associated marker of senescent chondrocytes using a cell-based ELISA. We describe the steps for primary chondrocyte culture and chemical induction of senescence. This protocol offers a high-throughput screening method to simultaneously quantify CSV across multiple samples with minimal cell input.

## Highlights

• Protocol to study CSV as a membrane-associated marker of senescence
• Steps for reliable induction of stress-induced cellular senescence
• Guidance on CSV quantification using a cell-based ELISA procedure

## Abstract (editor highlights)

Publisher’s note: Undertaking any experimental protocol requires adherence to local institutional guidelines for laboratory safety and ethics.

## Graphical abstract

**Figure:**
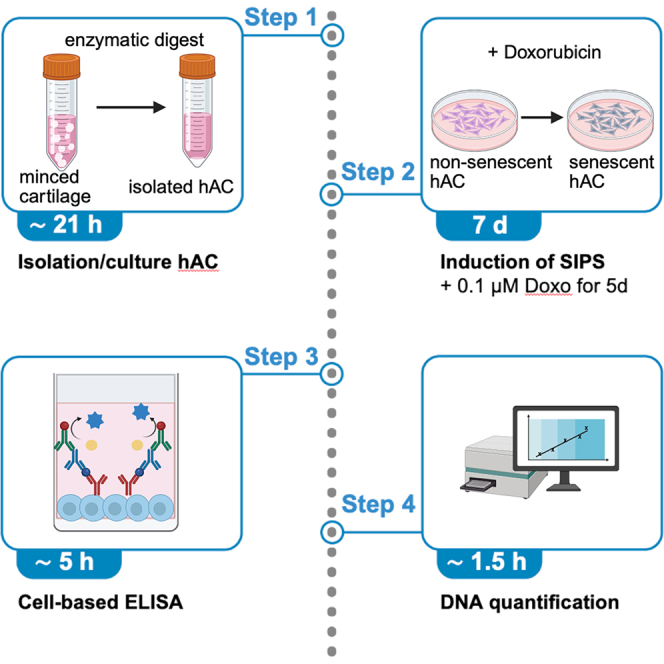

## Before you begin

Senescent cells play a key role in various degenerative diseases, including osteoarthritis (OA) – one of the most common musculoskeletal diseases world-wide. Due to increased oxidative stress and inflammation, senescent chondrocytes accumulate in the intra-articular cartilage, thereby contributing to tissue degeneration and thus the progression of OA. Consequently, targeting these dysfunctional cells has emerged as a promising strategy in OA therapy.^1^^,^^2^

To specifically identify and target senescent chondrocytes, membrane-associated markers that define senescent chondrocytes are essential. Previously, we reported elevated levels of cell surface vimentin (CSV) on osteoarthritic and particularly on senescent human articular chondrocytes (hAC).^3^

CSV has initially been described as a marker of cancer stem-like cells expressing epithelial-mesenchymal transition (EMT) markers.^4^ Furthermore, CSV was described on non-cancer cells, such as bone marrow-derived mesenchymal stem cells^5^ or apoptotic neutrophils.^6^ Despite growing interest in this mislocated intermediate filament, the role and biological function of CSV remain largely unclear. It has been speculated that CSV may act as an ‘eat-me’ signal for phagocytes and has also been associated with cell adhesion to specific substrates.^3^^,^^5^^,^^6^

In previous publications, CSV was detected by means of flow cytometry and immunofluorescence (IF) or immunocytochemistry (ICC) using a CSV-specific antibody [clone 84–1].^3^^,^^4^ While flow cytometric assessment of CSV requires high cell numbers, the ability to detect CSV on adherent cells enabled the development of a cell-based ELISA, which can be performed with relatively few cells. In previous studies, we applied this approach to quantify the terminal complement complex on primary cells and directly investigate potential inhibitors or activators of the complement cascade.^7^^,^^8^

### Innovation

Although flow cytometric analysis represents a suitable methodologic approach to assess the percentage of CSV-positive cells, the analysis requires a high cell count and is not suitable for a fast screening of the cell surface marker under different conditions. This protocol provides step-by-step guidance for quantifying CSV on isolated hAC or other cell types, with the possibility to directly test compounds or factors that influence vimentin externalization.

### Institutional permissions

**Timing: 1 h**

Human samples for hAC isolation were obtained from donors undergoing total knee joint replacement (arthroplasty) due to osteoarthritis. Informed consent was obtained from all patients according to the terms of the Ethics Committee of the University of Ulm (ethical approval number 353/18).

Prepare digest solutions for enzymatic chondrocyte isolation

1. Prepare sterile protease digest solution (needed for step 2).
  a. Use 1 g protease and dissolve in 500 mL DMEM to obtain a 0.2 % protease solution.
    ***Note:*** Use DMEM at room temperature to improve the solubility.
  b. Vortex until solution appears clear.
  c. Sterile filtrate the solution through a 0.2 μm filter.
  d. Prepare aliquots à 20 mL and freeze at −20°C.
    ***Note:*** Protease solution can be stored at −20°C for up to 6 months.
2. Prepare sterile collagenase digest solution (needed for step 3).
  a. Use 0.125 g collagenase and dissolve in 500 mL DMEM to obtain a 0.025 % collagenase solution.
    ***Note:*** Use DMEM at room temperature to improve the solubility.
  b. Vortex until solution appears clear.
  c. Sterile filtrate the solution through a 0.2 μm filter.
  d. Add 5 mL penicillin/streptomycin (final concentration 1x).
  e. Prepare aliquots à 20 mL and freeze at −20°C.
    ***Note:*** Collagenase solution can be stored at −20°C for up to 6 months.

## Key resources table

**Table:** 

REAGENT or RESOURCE	SOURCE	IDENTIFIER
**Antibodies**		

CSV monoclonal, clone 84-1	abnova	H00007431-M08 RRID:AB_3698143; https://www.antibodyregistry.org/AB_3698143
biotinylated goat anti-polyvalent	abcam	ab64264

**Chemicals, peptides, and recombinant proteins**		

2-phospho-L-ascorbic acid trisodium salt	Sigma-Aldrich	49752
3,3′,5,5′-Tetramethylbenzidine (TMB) Liquid Substrate	Sigma-Aldrich	# T0440-100ML
Antibody diluent	Agilent Dako	S080983-2
Bovine serum albumin (BSA)	Sigma-Aldrich	A3059
Collagenase (from Clostridium histolyticum)	Merck	C5138
DMEM	gibco	31885–025
DNA from calf thymus	invitrogen	15633019
Doxorubicin	Selleckchem	S1208
EDTA (Titriplex® III)	Sigma-Aldrich	1.08418
Fetal bovine serum (FBS)	PAN Biotech	P30-3031
Ham’s F12	PAN Biotech	P04-14550
Hoechst 33258	Fuka	33258
L-Glutamine	PAN Biotech	P04-80100
Paraformaldehyde	Thermo Scientific	047377.9M
Penicillin/Streptomycin (P/S)	PAN Biotech	P06-07100
Phosphate-buffered saline (PBS)	gibco	14190–094
Protease (from Streptomyces griseus)	Merck	P5147
Proteinase K (from Tritirachium album)	Sigma-Aldrich	P-2308
Streptavidin HRP solution	Agilent Dako	P039701-2
Sulfuric Acid 2N	R&D Systems	#895032
Tris (Trizma base)	Sigma-Aldrich	93362
Trizma-HCl (Tris-HCl) TRIS Hydrochloride	Carl Roth	9090.3
Trypsin/EDTA	PAN Biotech	P10-023100
Tween-20	Sigma-Aldrich	P1379

**Other**		

Tecan Reader M200 Pro	Tecan Austria GmbH	Ref. 30050303 SN 1111009133
Senescence-associated β-galactosidase staining kit	Cell signaling	# 9860

## Materials and equipment

**Table:** Chondrocyte culture medium

Reagent	Final concentration	Amount
DMEM (high glucose)	–	178 mL
Ham’s F12	–	178 mL
FCS	10%	40 mL
L-glutamine	0.5%	2 mL
Penicillin/Streptomycin	0.5%	2 mL
Ascorbic acid (stock: 5mg/mL)	10 μg/mL	800 μL
Total	–	500 mL

Cell culture medium can be stored at 4°C for up to four weeks. Needed for step 4 and following.

### PBS-buffered EDTA solution (10 mM)

• Dissolve 1.8612 g EDTA (Titriplex® III) in approx. 350 mL PBS.
• Use 10% NaOH and 1M NaOH to adjust pH (pH 7.2–7.4).
• After adjusting the pH, fill up to 500 mL with PBS to obtain a final concentration of 10 mM.

***Note:*** PBS-buffered EDTA solution can be stored at 4°C for up to 3 months. Needed for step 11.

### Proteinase K digest solution

• Dissolve 0.5 mg/mL Proteinase K in 30 mM Tris-HCl (pH 8).
• Prepare aliquots of 1 mL.

***Note:*** Proteinase K digest solution can be stored at −20°C for up to 6 months. Needed for step 29.

**Table:** TBS buffer (10x)

Reagent	Final concentration	Amount
Trizma-HCl	154 mM	24.23 g
NaCl	1.5 M	87.60 g
dH2O	–	800 mL
Total	–	1 L

• Stir buffer ON until the salts are completely dissolved.
• Use 10% NaOH and 1M NaOH to adjust pH to 7.6.
• After adjusting the pH, fill up to 1 L with H2O.

***Note:*** TBS Buffer can be stored at 4°C for up to 3 months.

### TBS-T buffer (1×)

• Use 100 mL 10x TBS and dilute with 900 mL dH2O to obtain a 1x TBS buffer.
• Add 200 μl Tween-20 to 200 mL TBS (1x) to obtain a final Tween-20 concentration of 0.1% (v/v).

***Note:*** Prepare fresh from the TBS stock buffer (10x). Needed for step 19 and following.

### Blocking buffer

• Use 2 mL 10x TBS and dilute with 18 mL dH2O to obtain a 1x TBS buffer.
• Add 1 g BSA to 20 mL 1x TBS buffer to obtain a 5% BSA solution.
• Mix rigorously.
• Prepare aliquots of 1.5 mL.

***Note:*** Blocking buffer ca be stored at −20°C for 6 months. Needed for step 14.

**Table:** TNE buffer

Reagent	Final concentration	Amount
Tris	10 mM	1.21 g
Na_2_EDTA · 2H_2_O	1 mM	0.37 g
NaCl	3 M	175.32 g
dH2O	–	700 mL
Total	–	1 L

• Use concentrated HCl to adjust the pH to 7.4.
• After adjusting the pH, fill up to 1 L with H2O.

***Note:*** TNE Buffer can be stored at 4°C for up to 3 months. Needed for step 30 and following.

## Step-by-step method details

### hAC culture

**Timing: 1.5 days (sample preparation: 2 h, pre-digest including washing: 1 h, digest: 16 h, seeding: 2 h)**

This initial section describes the procedures for obtaining primary hAC from clinical specimen, derived from osteoarthritis patients undergoing arthroplasty.

***Note:*** Perform all steps under sterile conditions.

1. Prepare cartilage pieces from macroscopically intact human knee cartilage (femoral condyles).
  a. Place resected human femoral condyles in a sterile kidney dish and rinse in PBS to remove blood contaminations.
  b. Cut macroscopically intact cartilage parallel to the bone to obtain thin flakes (thickness approx. 0.5–1 mm) and place in a conical 50 mL tube with sterile PBS. Fix the specimen at the bony part using surgical forceps and cut with a scalpel.
    ***Note:*** As scalpel blades, we use no. 11 or no. 23.
  c. Discard the specimen (bone, degenerated cartilage tissue) from the kidney dish and rinse with sterile PBS to remove non-cartilage tissue debris and blood.
  d. Place cartilage flakes into the kidney dish and cut in small pieces (approx. 0.5–1 x 1 mm) without compressing the cartilage.
    ***Note:*** Scalpel blades quickly become blunt due to the cutting on the metallic dish. Change the blades to avoid unnecessary compression of the tissue.
  e. Place cartilage pieces in a conical 50 mL tube and wash twice with sterile PBS by shaking and decanting the PBS.
2. Pre-digest cartilage pieces in protease solution.
  a. Decant the PBS and add 10–12 mL pre-warmed protease solution per gram of cartilage.
  b. Place conical 50 mL tube in a rotary wheel (e.g., hybridization oven) at 37°C for 30 min.
  c. Wash the cartilage pieces twice rigorously with 20 mL PBS to remove protease solution.
3. Digest cartilage pieces in collagenase solution.
  a. Decant the PBS and add 10–12 mL pre-warmed collagenase solution per gram of cartilage.
  b. Place conical 50 mL tube in a rotary wheel (e.g., hybridization oven) at 37°C ON.

**CRITICAL:** ON digest should not exceed 17 h.

4. Wash isolated hAC.
  a. Pour digested cartilage pieces on a cell strainer (40 μm) into a fresh conical 50 mL tube.
  b. Add 10 mL PBS to the digestion tube, rinse, and pour on the cell strainer to flush remaining cells.
  c. Centrifuge conical 50 mL tube at 400 g for 10 mins (RT) to pellet cells.
  d. Decant the digest solution and dissolve the cell pellet in 20 mL pre-warmed chondrocyte culture medium by gently pipetting up and down using a 10 mL serological pipette.
    ***Note:*** Instead of culture medium, DMEM supplemented with 1% PS can be used.
  e. Centrifuge conical 50 mL tube at 400 g for 10 mins to pellet cells.
  f. Decant the digest solution and dissolve the cell pellet in 20 mL pre-warmed culture medium by gently pipetting up and down using a 10 mL serological pipette.
5. Count and seed the cells for culture.
  a. Assess the cell count by manual or automated cell counting.
  b. Seed about 10,000 hAC per cm^2^ in pre-warmed chondrocyte culture medium on 75 or 175 cm^2^ flasks.
    ***Note:*** Freshly isolated hAC are considered at passage 0.
  c. Culture cells in an incubator at 37°C, 95% humidity, and 5% CO2.
    ***Note:*** Check adherence of isolated cells after 24 h and gently rinse with PBS to remove floating cells and remaining tissue debris.
6. Make sure cells are split at 80% confluency.
  a. Use Trypsin/EDTA (0.05%/0.02%) to detach hAC from cell culture plate. Apply for 10 min at 37°C.
  b. Seed the cells at a density of 5000 cells/cm^2^ for further expansion.
    ***Note:*** After isolation, cells usually need some time to start proliferation.
  c. Culture cells in an incubator at 37°C, 95% humidity, and 5% CO2 and change the chondrocyte culture medium twice a week.

### Induction of senescence in hAC

**Timing: 7 days**

This section provides a detailed protocol for inducing a stable stress-induced premature senescence (SISP) phenotype in hAC as previously reported.^9^

7. Use hAC at passages 1–2 and seed into cell flasks.
  ***Note:*** At passages >2, hAC lose their chondrogenic phenotype and undergo senescence in culture. This phenotypical alteration leads to externalization of vimentin and thus increased CSV levels (see expected outcomes section). If the assay is applied on a different cell type, we recommend to check this passage-dependent effect on CSV levels for the respective cell type.
  a. Use Trypsin/EDTA to detach hAC from cell culture plate. Apply for 10 min at 37°C.
  b. Count the cells and seed 4,500 cells/cm^2^ for the control cells and 5,500 cells/cm^2^ for the doxorubicin-treated group.
    ***Note:*** Only a small cell count is required for the cell-based ELISA. However, to obtain enough senescent cells for characterization, seeding on a 75 cm^2^ or even 175 cm^2^ flask is recommended, because the cells do not proliferate.
8. Add doxorubicin to reach a final concentration of 0.1 μM in the culture medium.

***Note:*** Doxorubicin can be added directly after seeding or the next day. To our experience the drug does not influence the adherence of the cells.

**Figure 1:**
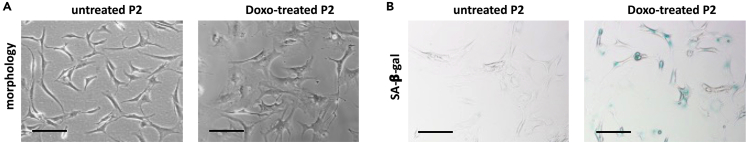
Brief confirmation of senescence in hAC (A) Visual confirmation of the senescent phenotype using a phase contrast microscope. Senescent hAC exhibit clear morphologic alterations, including an elongated and/or flattened cell shape. Moreover, confluency might be lower as compared to non-senescent cells due to the cell cycle arrest. (B) Additional confirmation of senescence by means of a senescence associated SA-β-Gal staining. Accumulation of SA-β-Gal and its enzymatic activity at a pH of 6 results in a bluish coloring of the cells. The scale bars represent 50 μm.
  b. Confirm senescence at least via a senescence-associated β-galactosidase (SA-β-Gal) staining (Figure 1B).
    ***Note:*** Cells should exhibit clear morphological changes. It is highly recommended to confirm senescence by at least a common SA-β-Gal staining and gene expression analysis (e.g., CDKN1A, CDKN2A). For further characterization of senescent hAC and the establishment of the doxorubicin model, please see our previous publication.^9^
    ***Note:*** In our experience, treatment with 0.1 μM doxorubicin can result in > 80% SA-β-Gal^+^ cells.^9^

9. Add doxorubicin for five consecutive days. On day five, double dose of doxorubicin can be applied.
  a. Check the cell viability and morphology each day by phase contrast microscopy.
    **CRITICAL:** Cells might change their morphology and become elongated or flat. If another cell type or cell line is used, the dosage and application time of doxorubicin should be adjusted. Loss of adhesion (roundish shape) and cell death indicates that the concentration of doxorubicin is too high and cells rather undergo apoptosis than senescence. As senescent cells are considered as apoptosis resistant, apoptosis needs to be avoided.^9^
  b. Add 0.1 μm fresh doxorubicin daily.
    ***Note:*** Doxorubicin has a reported half-life of approximately 10–20 h at 37°C in cell culture media. Although residual doxorubicin may remain in the medium after 24 h, a medium change between applications is not considered necessary for primary hAC under these conditions.
  c. Change the medium twice a week.
  d. Check confluency of cells and split control cells at 80% confluence if needed (see step 7).
    ***Note:*** Application of doxorubicin should quickly decelerate proliferation. Thus, it is very unlikely that doxorubicin-treated cells need to be split.
10. Confirm senescence of cells and use at day 7 or later.
  ***Note:*** The senescent phenotype should be stable for at least 2 weeks after doxorubicin application. However, this strongly depends on the cell type and needs to be confirmed.
  a. Check the cell viability and morphology by phase contrast microscopy (Figure 1A).

### Performance of the cell-based ELISA

**Timing: 1.5 days (seeding: 2 h, resting phase: ON, ELISA: 5.5 h)**

The following sections provides a stepwise protocol of the ELISA-based detection of CSV on hAC, starting with the seeding of the cells the day before.

***Note:*** The following steps should be performed using a laminar flow hood at least until step 13.

11. Detach cells from culture plate and count the cells.
  a. Remove culture medium from non-senescent (untreated) and senescent (doxorubicin-treated) cells and rinse with PBS.
  b. Detach hAC by adding 10 mM PBS-buffered EDTA and incubating for about 20 min at RT.
    ***Note:*** CSV can be degraded by trypsin as previously described.^3^ Although the detection of CSV will be performed at least 24 h after seeding, we recommend usage of 10 mM EDTA at this step, as we have not determined the time required to fully recover CSV.
  c. Add chondrocyte culture medium for trypsin inactivation and remove remaining adherent cells via a cell scraper.
  d. Centrifuge cell solution at 400 g for 10 mins.
  e. Discard the supernatant and resuspend cell pellet in culture medium.
  f. Count the cells manually or automated.
12. Seed cells on a 96 well plate.
  a. Dilute the cell suspension to 8000 cells/100 μL using culture medium and add 100 μL into each well of a 96 well plate.
    **CRITICAL:** Add an additional well of cells for the negative control in step 16. Spare the first two rows. Fill perimeter wells with PBS to further reduce evaporation during proteinase K digestion (step 29).
    ***Note:*** Dilution is not necessarily required. However, avoid pipetting too small volumes of the cell suspension and usage of small pipette tips (such as 10 μL or 20 μL) to reduce shear forces. Total volume should be about 100 to 150 μL.
    ***Note:*** Samples can be analyzed in duplicates to increase accuracy.
  b. Place culture plate into cell incubator and let cell rest for approx. 24 h (at least ON).
    ***Optional:*** CSV levels might be influenced by cellular stress or external stimuli, such as inflammatory cytokines. To screen for potential inducers of vimentin externalization, untreated cells can be exposed to, e.g., pro-inflammatory mediators or radical producers, the day after seeding. Incubation time depends on the cell type and concentration of the potential inducer. In the expected outcome section, we provide exemplary results after stimulating hAC with different stimuli for 24 h.
    **CRITICAL:** The antibody binding might be impaired by fixation of the cells. Therefore, steps 13 to 17 are executed on unfixed cells. Be careful and do not touch the cells on the well bottom with the pipette! Place the pipette tip on the well wall and let liquids flow in gently. It is advisable to frequently check the cell adherence during the assay. After step 18 (fixation) detachment of the cells is less likely but gentle pipetting is still recommended.
13. Wash cells twice with PBS.
  a. Discard cell culture medium by quickly turning the plate upside down over the sink.
  b. Add 300 μL PBS using a multichannel pipette.
  c. Discard PBS by quickly turning the plate upside down over the sink.
  d. Repeat steps 13 b and c.
  e. Gently tap the empty plate on paper towel once to remove remaining liquid.
14. Add 100 μL Blocking buffer to each well and let incubate for 1 h at 37°C, 95% humidity, and 5% CO2.
15. Discard Blocking Buffer by turning the plate upside down over the sink and gently tap the empty plate on paper towel once to remove remaining liquid.
16. Add 50 μL of the primary antibody (anti-CSV, clone 84–1) at a 1:1000 dilution (1 μg/mL in AB diluent) to each well and incubate for 2 h at 37°C, 95% humidity, and 5% CO2.

***Note:*** A negative control is recommended. The cells are incubated with 50 μL of AB diluent without primary antibody.

17. Wash cells three times with PBS, let PBS soak for 1 min (see step 13 a-e).
18. Fix cells with 4% paraformaldehyde (100 μL/well) for 15 min at RT.
19. Wash cells three times with TBS-T, let TBS-T soak for 1 min (see step 13 a-e).
20. Add 50 μL/well of the biotinylated goat anti-polyvalent solution and let incubate for 30 min at RT.

***Note:*** Other biotinylated secondary antibodies can be used. Although the direct use of an HRP-conjugated secondary antibody would save time, signal amplification through the biotin-based system yielded better results in our hands.

21. Wash cells three times with TBS-T, let TBS-T soak for 1 min (see step 13 a-e).
22. Add 50 μL/well of the HRP-coupled streptavidin solution and let incubate for 30 min at RT.
23. Wash cells three times with TBS-T, let TBS-T soak for 1 min (see step 13 a-e).
24. Add 70 μL/well of the TMB substrate solution and incubate at RT in the dark for max. 20 min.

***Note:*** Frequently control the color change and stop the reaction when the first well reaches a dark bluish color.

25. Transfer 50 μL of each well to a new plate using a multichannel pipette.
26. Add 50 μL stop solution to each reaction.

***Note:*** It is also possible to pre-fill the wells on the new plate with stop solution and directly add the reaction into the stop solution.

27. Directly measure the absorption at 450 nm after addition of stop solution using a microplate reader.

### DNA quantification for normalization

**Timing: 1.5 days (proteinase K digest: ON, DNA quantification: 1.5 h)**

The next steps are necessary to normalize the absorption of the cell-based ELISA to the cell number. This way, differences in cell numbers due to detachment during the assay or different proliferation potential can be subtracted.

28. Wash cells twice with PBS (see step 13 a-e).
29. Perform proteinase K digestion of cells.
  a. Add 100 μL/well proteinase K digest solution into wells containing cells and into three cell-free wells (controls).
  b. Incubate overnight at 56°C with gentle shaking (∼200 rpm) in a humid chamber.

***Note:*** Place the well plate on paper towels soaked with water in a metal instrument tray with lid to create a humid chamber and avoid evaporation of the digest solution.

30. Fill up the volume in all wells to 120 μL/well using TNE buffer and gently mix by pipetting up and down.

***Note:*** Control the volume in all wells as evaporation might have reduced the volume.

31. Transfer digest solution into 1.5 mL reaction tubes and centrifuge at 3000 g for 5 min. Carefully transfer the supernatant to a fresh tube without disturbing the pellet.
32. Prepare fresh Hoechst solution.
  a. Add 1 μl Hoechst (1mg/mL) to 5 mL TNE.

***Note:*** Always prepare solution freshly.

**Figure 2:**
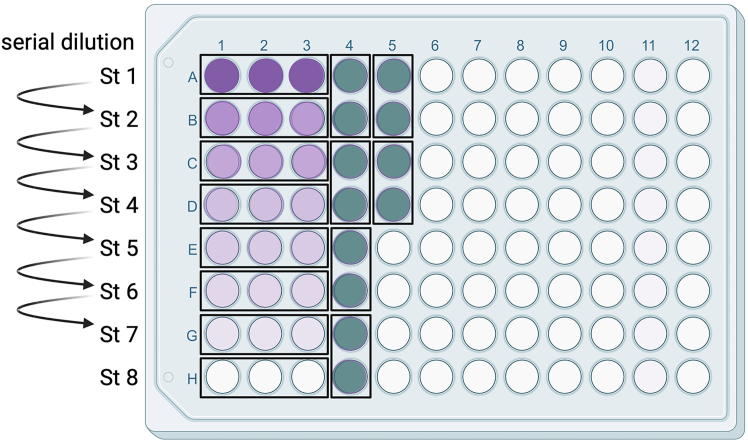
Schematic illustration of the pipetting scheme Standard (St; purple) solution is analyzed in triplicates and samples (green) in duplicates.

33. Prepare a DNA standard in triplicates (see Figure 2).
  a. Use a fresh black bottom 96 well plate.
  b. Add 999 μL TNE buffer in wells A1, A2, and A3.
  c. Add 50 μL TNE buffer in wells B-H1, B-G2, and B-G3.
  d. Add 1 μL of 1 mg/mL DNA stock solution to 999 μL of TNE in wells A1, A2, and A3.
  e. Perform a serial dilution by transferring 50 μL of row A to 50 μL TNE in row B. Mix rigorously by pipetting up and down.
  f. Repeat serial dilution until row G.
  g. Take 50 μL of row G and discard.

***Note:*** Use multichannel pipette for the preparation of the DNA standard.

**Figure 3:**
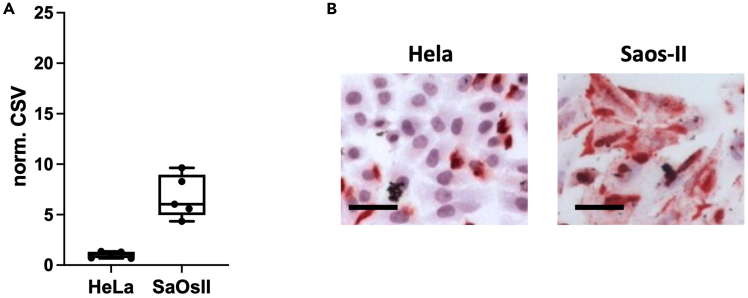
Validation of the cell-based CSV ELISA using cancer cell lines (A) Quantification of the CSV levels on HeLa (low CSV positive) and SaOsII (high CSV positive). Absorption levels (OD_450_) were normalized to the results of the DNA quantification. Data are presented as box plots with individual data points. The median is shown, and whiskers indicate the minimum and maximum values. (B) Exemplary ICC staining of CSV on HeLa and SaOs-II cells. The scale bars represent 50 μm.

**Figure 4:**
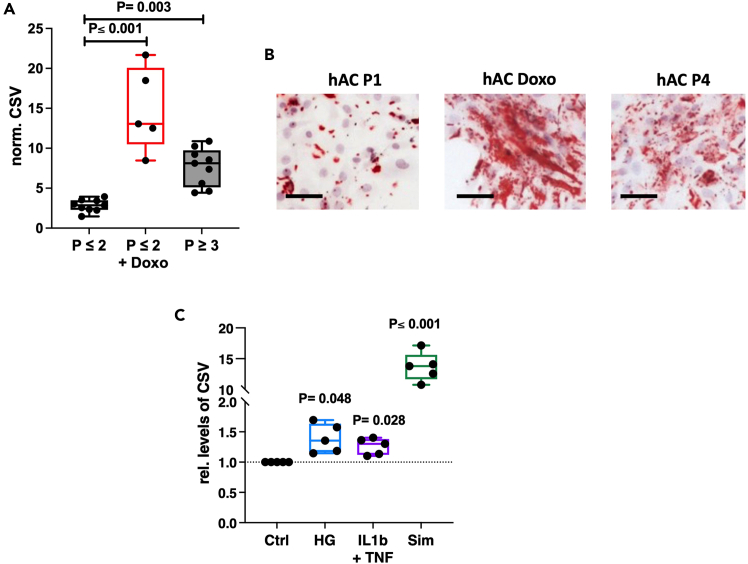
Quantification of CSV on senescent hAC and investigation of potential inducers of vimentin externalization (A) Quantification of the CSV levels on low passage (P ≤ 2), doxorubicin-treated (P≤ 2 + Doxo), and high passage (P ≥ 3) hAC using the cell-based CSV ELISA. Absorption levels (OD_450_) were normalized to the results of the DNA quantification. (B) Exemplary ICC staining of CSV on hAC at passage 1 (hAC P1), after doxorubicin treatment (hAC Doxo), and at passage 4 (hAC P4). The scale bars represent 50 μm. (C) Quantification of CSV levels on hAC treated for 24 h with cartilage homogenate (HG), a cytokine cocktail containing IL-1b and TNF (10 ng/mL each; IL1b + TNF), or Simvastatin (10 μM; Sim) relative to CSV levels on untreated hAC (Ctrl). Absorption levels (OD_450_) were normalized to the results of the DNA quantification. Data are presented as box plots with individual data points. The median is shown, and whiskers indicate the minimum and maximum values. Statistical analysis: One-way ANOVA with Sidak post-hoc test.

Standards (St)

DNA from calf thymus, stock solution: 1 mg/mL.

**Table:** 

St1:	999 μL TNE buffer + 1 μL DNA stock solution	(1 μg/mL)
St2:	1:2 dilution of St1	(0.5 μg/mL)
St3:	1:2 dilution of St2	(0.25 μg/mL)
St4:	1:2 dilution of St3	(0.125 μg/mL)
St5:	1:2 dilution of St4	(0.0625 μg/mL)
St6:	1:2 dilution of St5	(0.03125 μg/mL)
St7:	1:2 dilution of St4	(0.015625 μg/mL)
St8:	TNE buffer	(0.0 μg/mL)

34. Transfer 50 μL of the digest solution in duplicates on the black bottom plate next to the standard (starting at A4).
35. Add 50 μL of 2x Hoechst solution to each well and incubate for 20 min at RT in the dark.
36. Measure the fluorescence intensity in the microplate reader at an excitation of 356 nm and an emission of 465 nm.
37. Normalize the results of the cell-base ELISA (OD_450_) to the DNA content of the respective well (normalized CSV = OD_450_/DNA fluorescence).

## Expected outcomes

To validate the cell-based ELISA, the assay was first applied on the cancer cell lines HeLa (low CSV levels) and SaOs-II (mediate/high CSV levels). Additional ICC staining was performed to visualize the CSV levels on the cell lines (Figures 3A and 3B).

The CSV ELISA was primarily established to investigate CSV on hAC as a membrane-associated marker of senescence. We previously reported that the presence of CSV is significantly increased on senescent (doxorubicin-treated) hAC as well as on high-passage hAC, due to replicative stress-induced senescence during in vitro cultivation.^3^ CSV quantification via the cell-based ELISA confirmed elevated CSV levels on doxorubicin-treated and high-passage hAC, which was also in line with the staining intensities after ICC (Figures 4A and 4B). In our hands, normalized CSV levels are typically ∼3–7-fold higher in doxorubicin-treated and high-passage hAC compared with low-passage controls.

To validate the suitability of the assay for investigating potential inducers of vimentin externalization, hACs were exposed to different factors starting the day after seeding (following step 12). As cytokines and matrix-derived damage-associated molecular factors are considered as potential triggers of cellular stress and senescence in OA, hAC were exposed to homogenized cartilage (HG)^7^ and a cytokine cocktail containing 10 ng/mL each of interleukin 1b (IL-1b) and tumor necrosis factor (TNF) for 24 h. These conditions resulted in a moderate increase of CSV levels (Figure 4C). In accordance with our previous study,^3^ 10 μM Simvastatin induced a strong externalization of vimentin, resulting in high CSV levels relative to untreated hAC (Figure 4C).

We conclude that the assay is suitable to assess CSV on primary hAC but may also be used for other cell types or cell lines to determine the membrane-associated marker. Furthermore, the assay allows rapid screening of different factors to evaluate their impact on vimentin externalization.

## Limitations

One limitation of the assay is the considerable time investment required, which may not be justified when analyzing only a small number of samples. In addition, inter-assay variability can be higher than that observed with flow cytometric CSV detection, due to the overall complexity of the procedure and potential deviations introduced at individual steps. Compared to flow assays, data on individual cells will not be available. Furthermore, no established procedure exists to correct for assay-specific sources of variation, such as differences in antibody incubation times, due to the absence of a concentration standard. Nevertheless, the advantages of the assay outweigh these limitations, as it enables simultaneous screening of multiple samples and experimental conditions with lower cell input compared to the conventional flow cytometry-based approach.

## Troubleshooting

### Problem 1

Bacterial or yeast contamination in the isolated chondrocytes.

### Potential solution

• First, it is recommended to improve overall cleanliness during chondrocyte isolation (e.g., use autoclaved instruments and disinfect hands frequently). Identify potential sources of contamination by carefully inspecting the digest solutions after each step (e.g., turbidity or yellowish discoloration of the digest solution after incubation at 37°C indicates possible contamination).
• Add penicillin/streptomycin (1×) to the protease digest solution.
• In case of yeast contamination, amphotericin (up to 2.5 μg/mL) can additionally be added to both the digest solution and the culture medium. It is recommended to remove amphotericin after the first passage to avoid long-term exposure to the antifungal agent, which may affect cell function, particularly proliferation.

### Problem 2

Low number of senescent cells after doxorubicin treatment.

### Potential solution

• If senescence was determined by means of a ß-galactosidase assay, please refer to the manufacturers troubleshooting and make sure that all decisive steps have been conducted accordingly, in particularly the pH of the galactosidase solution. The incubation time may be increased for several hours to improve staining intensity.
• Optimize the concentration and duration of doxorubicin treatment. Note that increased doxorubicin concentrations may induce apoptotic cell death (see^9^).
• Doxorubicin-mediated senescence is characterized by a strong morphological change (spreading) of the cells. Confluence of > 80 % might prevent spreading of the cells and thus induction of senescence. Therefore, reducing the seeding confluency to 60-70% might improve the outcome.

### Problem 3

There is no clear difference in the CSV signal (absorbance) between non-senescent and senescent cells, due to unexpectedly high CSV levels in control groups.

### Potential solution

• First, it is recommended to include HeLa (or any other low CSV-positive cell line) and SaOsII (or any other high CSV-positive cell line) in the assay as negative and positive control.
• The primary antibody might non-specifically bind to intracellular vimentin. Please ensure that cells have no contact to detergents (tween, triton-X) before the fixation (step 18). Afterwards, TBS-T can be used to wash cells.
• Primary cells, particularly chondrocytes, are prone to spontaneous senescence during in vitro culture (see Figures 4A and 4B). Additional factors, including suboptimal quality of the starting material, mechanical stress incurred during cartilage sectioning, and elevated shear forces experienced during pipetting of the cell suspension, can further exacerbate the induction of senescent phenotypes within the culture.

### Problem 4

DNA quantity is lower than the standard curve.

### Potential solution

• Please note that there might be differences due to the method of cell counting (manual or automated) or proliferation capacities of cells (especially in non-proliferating senescent cells), which might require further adjustments of the cell number used.
• It is recommended to increase the cell number either for all groups or only for those with impaired proliferation, such as senescent cells. Since CSV quantification is normalized to DNA content, seeding densities or cell numbers do not need to be identical between donors or experimental groups. However, it should be taken into account that high confluency and extracellular matrix production may potentially impair antibody accessibility to the antigen.

## Resource availability

### Lead contact

Further information and requests for resources and reagents should be directed to and will be fulfilled by the lead contact, Jana Riegger (jana.riegger@uni-ulm.de).

### Technical contact

Technical questions on executing this protocol should be directed to and will be answered by the technical contact, Jana Riegger (jana.riegger@uni-ulm.de).

### Materials availability

This study did not generate new unique reagents.

### Data and code availability

This study did not generate/analyze any datasets/code.

## Acknowledgments

We would like to thank Natalie Braun for her excellent technical support during the establishment of the assay. This study was supported by the Bausteinprogramm of the Medical Faculty, 10.13039/501100008977University of Ulm.

## Author contributions

J.R. established the protocols, performed the experiments, and analyzed the data; J.R. and H.G. wrote and edited the manuscript; and J.R. acquired the funding.

## Declaration of interests

The authors declare no competing interests.
